# Testing the Reproducibility of Multiple Displacement Amplification on Genomes of Clonal Endosymbiont Populations 

**DOI:** 10.1371/journal.pone.0082319

**Published:** 2013-11-27

**Authors:** Kirsten Maren Ellegaard, Lisa Klasson, Siv G. E. Andersson

**Affiliations:** Department of Molecular Evolution, Cell and Molecular Biology, Science for Life Laboratory, Biomedical Centre, Uppsala University, Uppsala, Sweden; University Of Montana - Missoula, United States of America

## Abstract

The multiple displacement amplification method has revolutionized genomic studies of uncultured bacteria, where the extraction of pure DNA in sufficient quantity for next-generation sequencing is challenging. However, the method is problematic in that it amplifies the target DNA unevenly, induces the formation of chimeric reads and also amplifies contaminating DNA. Here, we have tested the reproducibility of the multiple displacement amplification method using serial dilutions of extracted genomic DNA and intact cells from the cultured endosymbiont *Bartonella australis*. The amplified DNA was sequenced with the Illumina sequencing technology, and the results were compared to sequence data obtained from unamplified DNA in this study as well as from a previously published genome project. We show that artifacts such as the extent of the amplification bias, the percentage of chimeric reads and the relative fraction of contaminating DNA increase dramatically for the smallest amounts of template DNA. The pattern of read coverage was reproducibly obtained for samples with higher amounts of template DNA, suggesting that the bias is non-random and genome-specific. A re-analysis of previously published sequence data obtained after amplification from clonal endosymbiont populations confirmed these predictions. We conclude that many of the artifacts associated with the use of the multiple displacement amplification method can be alleviated or much reduced by using multiple cells as the template for the amplification. These findings should be particularly useful for researchers studying the genomes of endosymbionts and other uncultured bacteria, for which a small clonal population of cells can be isolated.

## Introduction

While the list of sequenced genomes has increased dramatically with the advent of next-generation sequencing technologies, a severe limitation is the requirement for a relatively high quantity of DNA for library preparation. Such quantities are readily obtained for bacteria which can be cultured in the laboratory, however the majority of environmental bacteria are uncultured [1]. Additionally, many of these bacteria are found in complex environments with a large diversity of microorganisms (e.g. soil), or in very low density (e.g. obligate endosymbionts), making it practically impossible to obtain high quantities of DNA from clonal populations of bacterial cells. 

One possibility for accessing the uncultured majority is to use the Multiple Displacement Amplification (MDA) method [2] to increase the quantity of DNA in the sample. The MDA method is based on the use of the Phi29 polymerase, which amplifies template DNA from random hexamer primers. The strand-displacement property of the Phi29 polymerase makes newly synthesized DNA accessible for new polymerase molecules, resulting in a continuous amplification reaction. With the aid of the MDA method, DNA can be amplified from as little as a single cell and the resulting material is usually sufficient for sequencing by next-generation sequencing technologies [3,4].

The MDA method has been used for several different applications. For example, it has been used as a pre-PCR step to ensure the availability of sufficient template DNA (e.g. 5,6), in metagenome projects of environmental bacteria in low abundance [7-9], and in clinical studies where the material of investigation is limited [10-12]. The MDA method has also been used in *de novo* genome sequencing projects of cells isolated from clonal bacterial populations [13-20]. Finally, MDA is becoming increasingly popular for single-cell genome amplification of environmental samples, sometimes in combination with metagenomic data [3,21-25]. 

The main concerns related to the use of MDA in these various applications are uneven amplification of the genome, formation of chimeric sequences and amplification of contaminating DNA [2]. The amplification bias is considered to be random [4,7,11,26-29] since independent MDA reactions on the same DNA template do not result in the same regions being over- and underrepresented. However, the genome sequencing of two bacterial endosymbionts from the genus *Wolbachia* indicated that some regions of the genome were consistently more highly amplified than others [17]. Since the amplification of DNA in these genome projects was performed using MDA on an unknown number of cells, we set out to investigate whether the amplification bias is related to the amount of template DNA. 

For this purpose, we have applied the MDA method to extracted genomic DNA and cells of the endosymbiont *Bartonella australis*, which unlike most endosymbionts can be cultured in the laboratory. The 1.6 Mb genome of *B. australis* has been sequenced previously from non-amplified DNA [30], which enables us to use this data as a control. After re-sequencing the *B. australis* genome using several independently amplified samples, we investigated the amplification bias, the percentage of chimeric reads, the genomic recovery, the effect on *de novo* genome assembly and the level and severity of contamination. We show that all these processes are affected by the amount of template DNA and we discuss the utility of the MDA method for whole genome sequencing projects of endosymbiont populations.

## Results

### Samples and sequencing

We re-sequenced *B. australis* strain NH1, for which a closed reference genome had previously been produced in our laboratory [30]. The re-sequencing was performed with the Illumina sequencing technology after amplification of the DNA with the Multiple Displacement Amplification method (MDA), using a standard commercial kit (Repli-G midi, Qiagen). Template material for the MDA reaction consisted of intact cells or extracted genomic DNA of *B. australis*, prepared in serial dilutions as detailed in (Table 1 and 2). We refer to the amplified DNA samples as “cells1-7” and “gDNA1-8” to indicate the source of template material, with the numbers indicating the relative dilutions of the sample. 

**Table 1:** Preparation of MDA samples from *B. australis* cells.

Sample	Dilution	Nb. of cells^a^	PCR^b^ locus: *batR*	PCR locus: *gltA*	PCR locus: 16S^c^
Cells 1	Undiluted	Lawn	+	+	+
Cells 2^d^	10^1^	Lawn	+	+	+
Cells 3^d^	10^2^	Many	+	+	+
Cells 4^d^	10^3^	2,6	+	+	+
Cells 5^d^	10^4^	0,2	-	+	(+)
Cells 6	10^5^	NA	-	-	(+)
Cells 7	10^6^	NA	-	-	(+)

^a^ Three plates were made from each dilution, where the samples cells4-5 yielded plates with colonies that could be reliably counted. Sample 6 and 7 had no colonies. The numbers were extrapolated to estimate the cell quantity in 3 μl template.

^b^ The PCR reactions were performed after the MDA reaction.

^c^ The 16S rRNA primers were universal, and yielded PCR products for all samples. Sanger sequencing showed that the samples cell 1-4 contained *B. australis*, whereas the samples cells 5-7 contained *Delftia acidovorans*.

^d^ Samples selected for sequencing

**Table 2:** Preparation of MDA samples from *B. australis* extracted DNA.

Sample	Amount of template DNA(ng)^a^	PCR^b^ locus: *batR*	PCR locus: *gltA*	PCR locus: 16S^c^
gDNA1^d^	34	+	+	+
gDNA2	3.4	+	+	+
gDNA3	0.34	+	+	+
gDNA4	0.034	+	+	+
gDNA5^d^	0.0034	+	+	+
gDNA6^d^	0.00034	+	+	+
gDNA7^d^	0.000034	+	+	+
gDNA8^d^	0.0000034	-	+	+

^a^ Only sample gDNA1 was measured, the following concentrations were estimated by extrapolation, according to the dilution

^b^ The PCR reactions were performed after the MDA reaction.

^c^ The 16S primers were universal, and yielded PCR products for all samples. Sanger sequencing confirmed the presence of *B. australis* in sample gDNA7-8.

^d^ Samples selected for sequencing

 The presence of the *B. australis* genome in each MDA sample was first tested by PCR on aliquots of the MDA samples, using primers specific for the *batR, gltA* and 16S rRNA genes. The PCR reactions with the universal 16S rRNA primers were positive for all MDA samples. To distinguish between *B. australis* and potential contaminants, these PCR products were sequenced. All MDA samples from the dilution series of genomic DNA yielded the expected 16S rRNA sequence from *B. australis* (Table 2). In contrast, for samples "cells5-7" (Table 1), the 16S rRNA sequences were identical to *Delftia acidovorans*, which has previously been identified as a contaminant in MDA samples [16,21,31]. Taken together with the PCR results from the *B. australis*-specific primers (*batR* and *gltA*), the screening of the MDA samples indicated partial presence of the *B. australis* genome in sample "cells5" and "gDNA8", and absence (or very limited presence) of the *B. australis* genome in sample "cells6-7".

 In the dilution series of genomic DNA, the sample with the lowest amount of template DNA (sample “gDNA8”) was estimated to contain around 3 femtogram of DNA, which is comparable to the amount of template DNA in single-cell samples. In the dilution series of intact cells, the number of template cells was roughly estimated by plate colony count (Table 1). Sample “cells5” produced colonies on all plates while the next sample in the dilution series, sample “cells6”, which contained a ten-fold lower amount of cells, failed to produce colonies in any of the three replicate platings. Therefore, we estimate that the sample “cells5” contained less than 10 cells, and was roughly comparable to a single-cell sample. 

 Based on these results, we selected 10 MDA samples for sequencing: Four samples were taken from the dilution series of intact cells (cells2-cells5) (Table 1) and five samples were taken from the dilution series of genomic DNA (gDNA1 and gDNA5-gDNA8) (Table 2). Thus, the MDA samples selected for sequencing differed in the amounts of template DNA by more than four orders of magnitude. After sequencing, we obtained approximately 1 million reads for the samples with the lowest amount of template DNA (mean coverage 100x) and around 1.5 million reads for all other samples (mean coverage > 200x) (Table S1). As an independent control of the distribution of sequence reads across the genome in unamplified DNA, we also included a previously published Illumina dataset from the genome project of *B. australis* in our analysis (mean coverage 136x) [30] (Table S2). To enable comparisons of the pattern of amplification between samples, we normalized the data for each sample to have the same mean coverage as the unamplified control sample sequenced in this study.

### Amplification bias in the *B. australis* genome

#### The magnitude of the amplification bias is dependent on the quantity of template DNA

**Figure 1:**
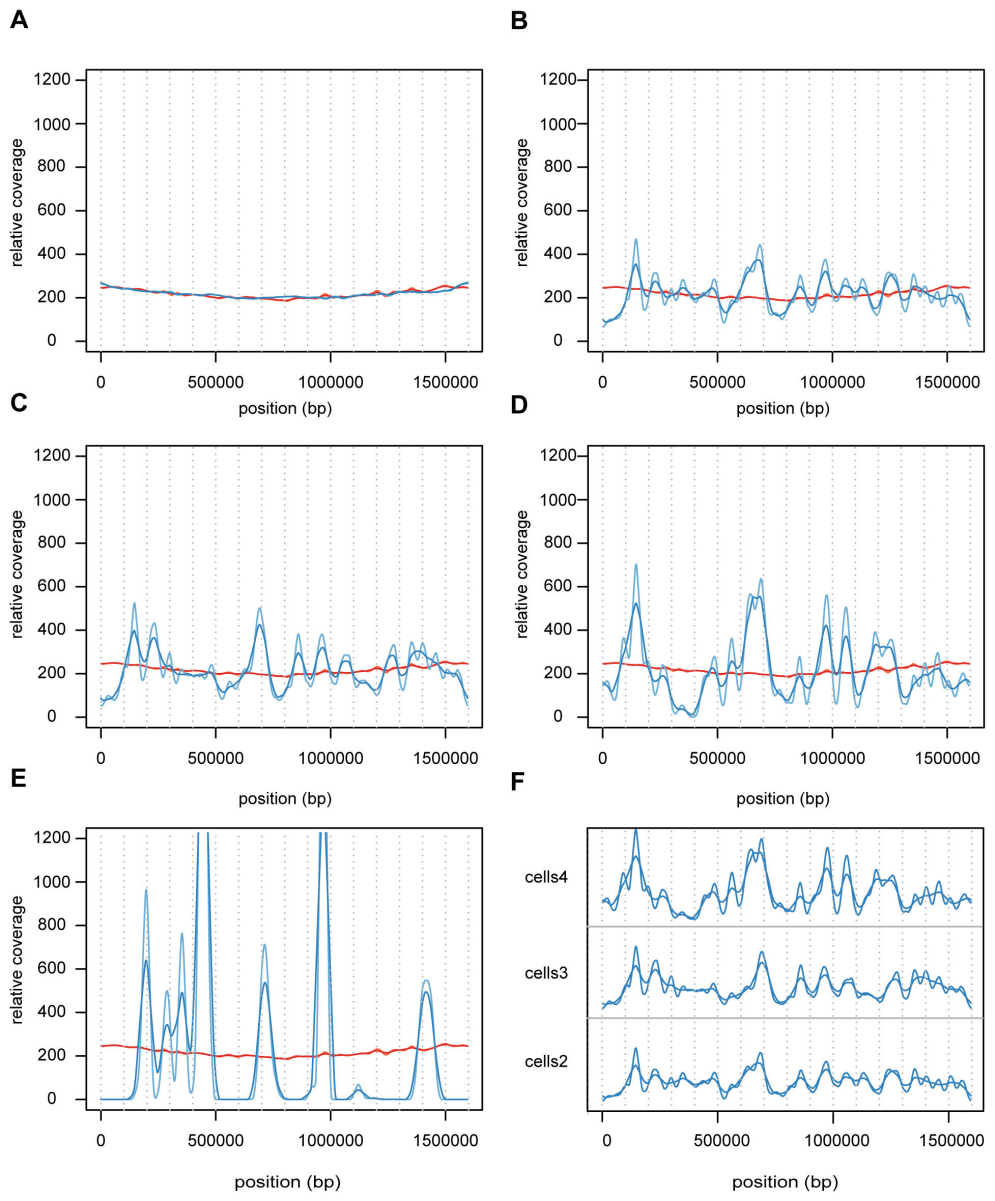
Concentration-dependent bias in read coverage of MDA samples obtained from bacterial cells. The coverage of sequence reads across the *B. australis* genome is shown for Illumina sequences generated from (**A**) the unamplified DNA from the previously published *B. australis* genome project and (**B**-**E**) the re-sequencing of the *B. australis* genome from MDA samples. Four different dilutions of bacterial cells were used as templates for the MDA reaction: (**B**) "cells2", (**C**) "cells3", (**D**) "cells4" and (**E**) "cells5", as detailed in Table 1. For each plot, the coverage of the unamplified control obtained from the re-sequencing of *B. australis* in this study is shown in red, and the sample for comparison in blue (two shades, corresponding to two moving average window sizes of 300 bp and 500 bp). The mean coverage of all samples was scaled to be the same as the control sample. (**F**) The coverage of samples “cells3” and “cells4” were scaled to have the same mean coverage as sample “cells2”, and plotted in the same window to facilitate visualization.

The coverage of sequence reads across the *B. australis* genome was even for both the unamplified control sample sequenced in this study and the previously published Illumina dataset (Figure 1A). All MDA samples displayed a bias in the coverage of sequence reads compared to the unamplified control samples, the magnitude of which was inversely related to the quantity of starting material (Figure 1B-E). Thus, the coverage was several orders of magnitude higher in the over-amplified regions in samples with the lowest quantity of DNA template compared to samples with higher quantities of DNA template. The same trends were observed irrespectively of whether cells (Figure 1) or genomic DNA (Figure S1) was used as the template for the MDA reaction.

**Figure 2:**
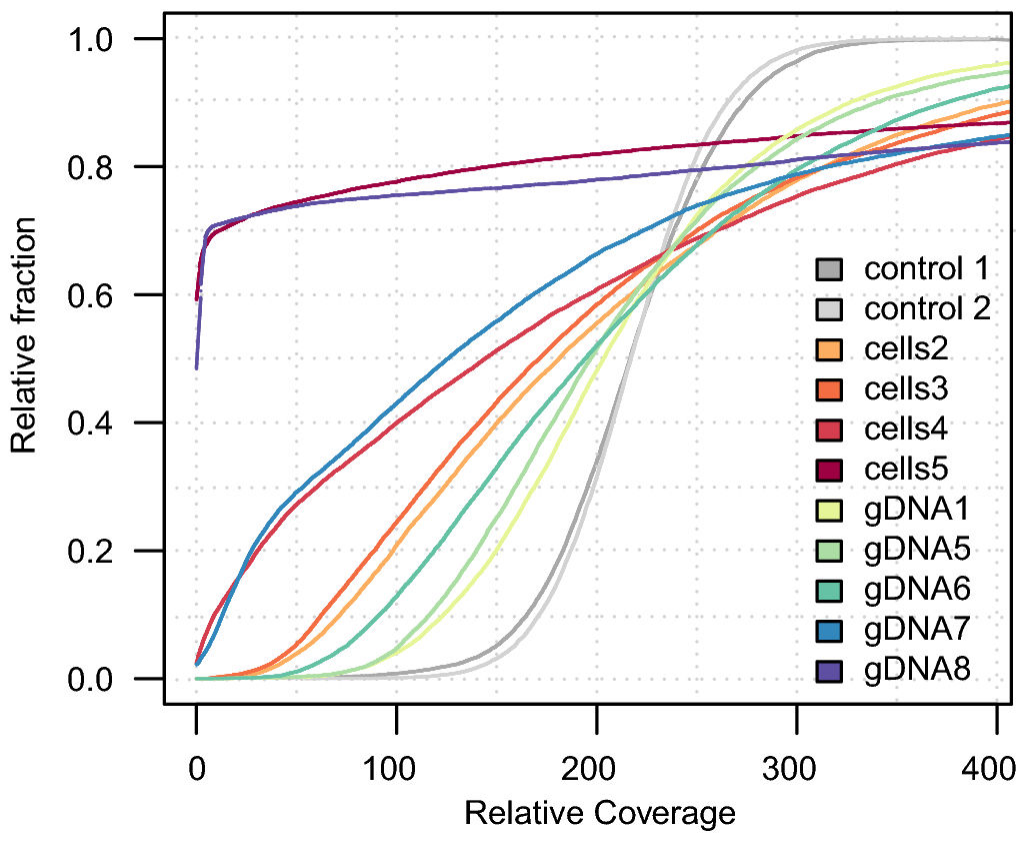
Cumulative read coverage distributions of the MDA samples. The graph displays the relative fraction of 100 bp windows with a mean coverage below or equal to the coverage given on the x-axis. Sample "control 1" refers to the unamplified Illumina data set obtained from the re-sequencing of the *B. australis* in this study (dark grey). Sample "control 2" refers to the unamplified Illumina data set obtained from the previously published *B. australis* genome (light grey). All samples were scaled to have the same mean coverage as sample "control 1".

 To visualize the differences in the magnitude of amplification bias, we plotted the cumulative coverage distributions for each sample (Figure 2). Samples with a minimal bias in coverage are expected to show a curve with a steep increase, reflecting the low variation in coverage across the genome. Indeed, the unamplified DNA as well as the samples with the highest amount of template DNA, “gDNA1” and “gDNA5”, showed a sharp increase near the average coverage value on the x-axis. In contrast, samples with a strong bias in coverage are expected to display a flat curve since a substantial fraction of the genome contains a very low coverage while other fractions of the genome contain a very high coverage. As expected, samples with the lowest quantity of template DNA, “cells5” and “gDNA8”, displayed nearly flat curves (Figure 2), consistent with an extremely biased distribution of sequence reads across the *B. australis* genome. 

#### The amplification pattern is non-random

To facilitate a direct comparison of the amplification patterns, we scaled the datasets “cells3” and “cells4” to have the same mean coverage as the sample “cells2”, and plotted the amplification curves on top of each other (Figure 1F). Visual inspection of the amplification patterns for these three samples indicated that most coverage peaks and valleys were located at approximately the same positions along the reference genome although the samples differed a 100-fold in the amount of template used for the MDA reaction (Figure 1F). The amplification pattern was similar although not identical when genomic DNA was used as the template, suggesting that the bias is determined mainly by the DNA sequence itself. 

**Figure 3:**
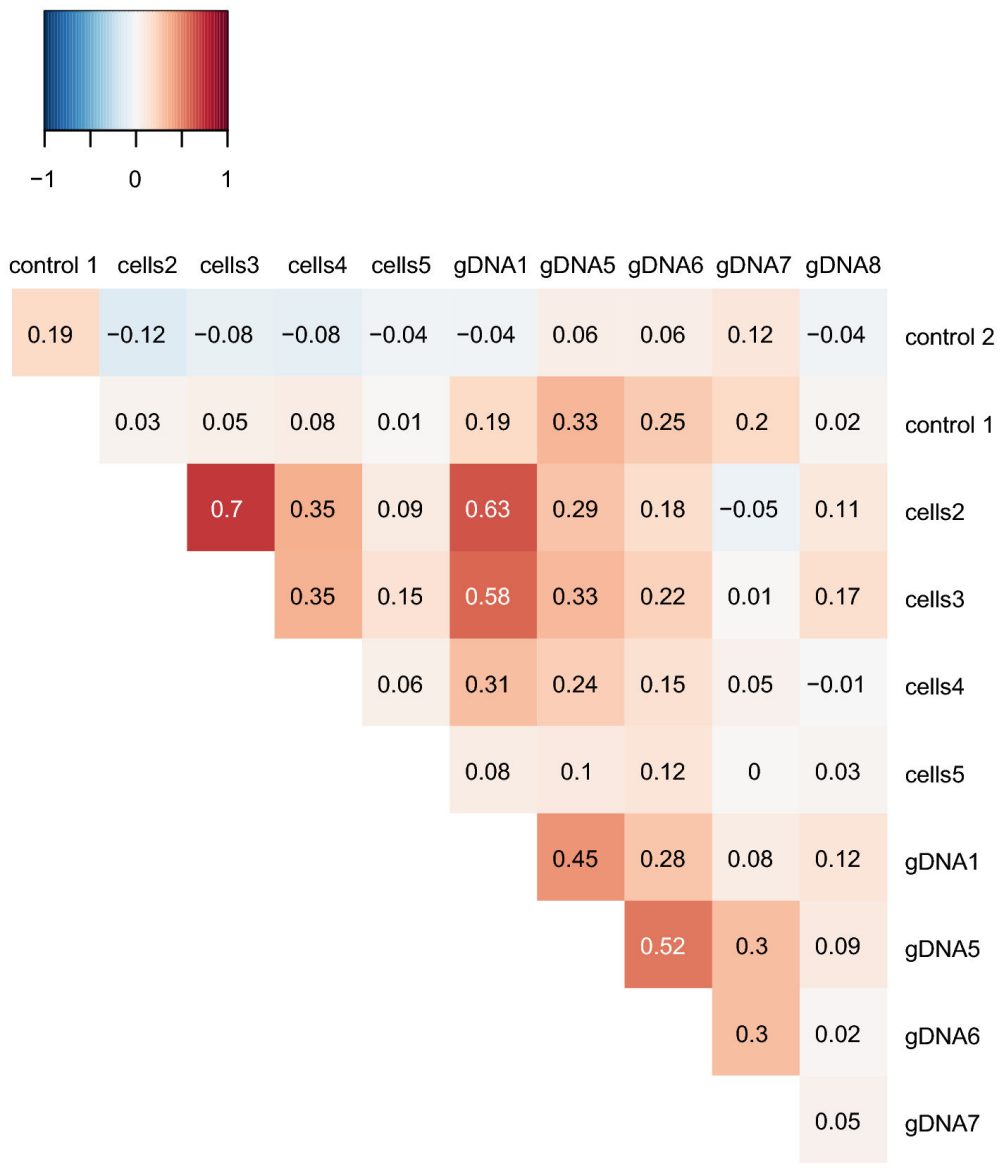
Heat map of Kendalls tau correlation coefficients. Pairwise correlations between the coverage patterns (averaged in 1 kb windows) in Illumina data sets of MDA samples taken from dilution series of intact bacterial cells (cells2-cells5) and genomic DNA (gDNA1-gDNA8), as detailed in Tables 1 and 2, respectively. Sample "control 1" refers to the unamplified Illumina data set obtained from the re-sequencing of the *B. australis* in this study. Sample "control 2" refers to the unamplified Illumina data set obtained from the previously published *B. australis* genome.

 To evaluate the similarity in patterns between samples, we calculated the correlation of coverage in 1000 bp windows between all possible pairwise combinations of samples using Kendalls Tau (Figure 3), which is a measure of the correlation between samples that do not follow a normal distribution [32]. Basically, all datapoints in each sample were ranked and the agreement between the ranking orders of two samples at a time was calculated. For example, if region A has a higher coverage than region B in both samples, the ranks are in agreement. We found the strongest correlations in amplification bias pattern between samples with similar quantities of template DNA. For example, sample “gDNA5” had a slightly higher correlation to sample “gDNA6” than to “gDNA1”. The highest correlation value was between the samples "cells2" and “cells3”, which were prepared from the largest number of cells of the samples analyzed here (see Figure 1F for a comparison of the patterns). 

**Figure 4:**
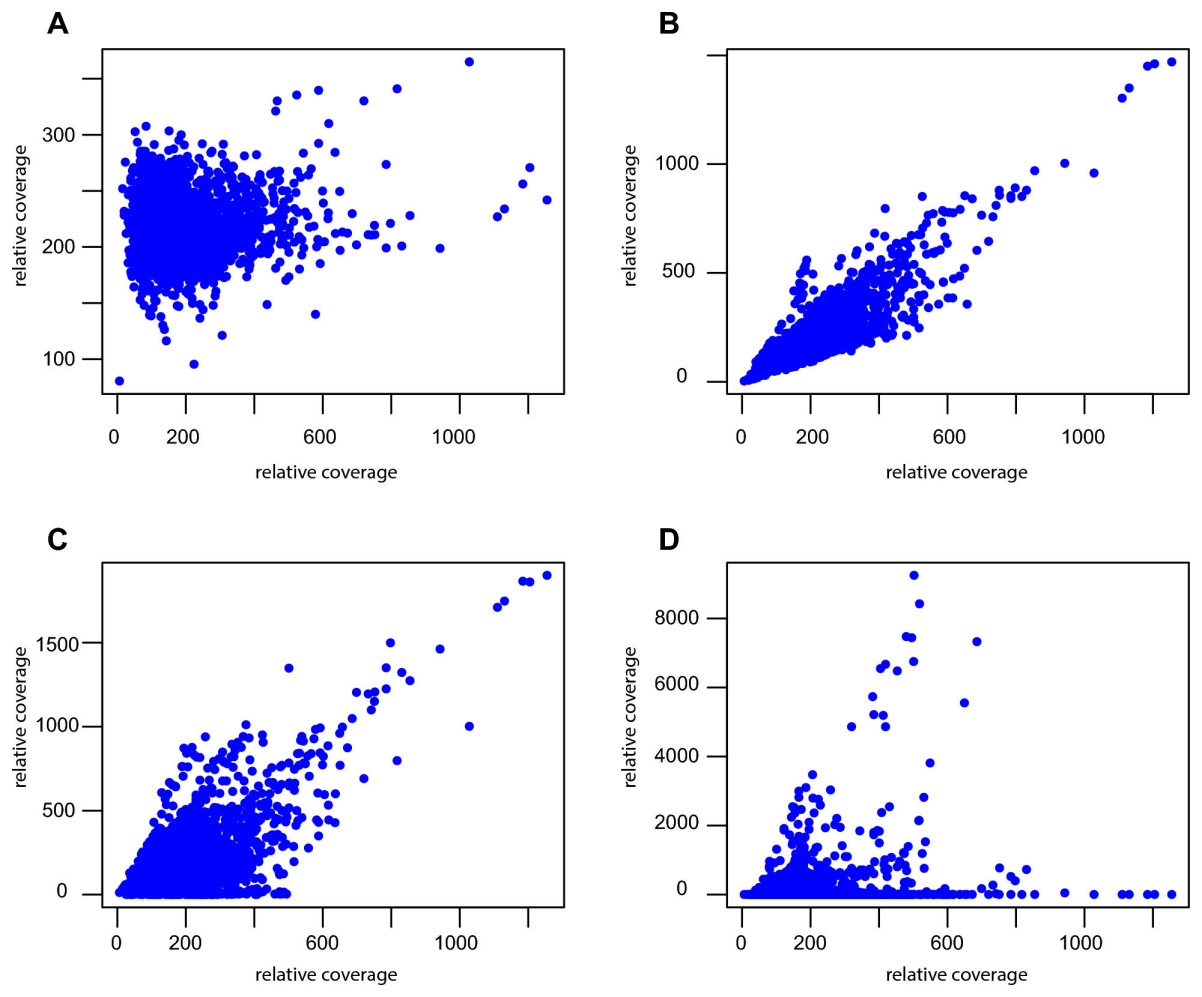
Coverage correlation plots. Pairwise comparisons of the coverage of sequence reads (averaged in 1 kb windows) across the *B. australis* genome in the Illumina data sets of MDA samples taken from a dilution series of intact bacterial cells, as detailed in Table 1. (**A**) "cells2" versus "control 1", (**B**) "cells2" versus "cells3", (**C**) "cells2" versus "cells4", (**D**) "cells2" versus "cells5". Sample "control 1" refers to the unamplified Illumina data set obtained from the re-sequencing of the *B. australis* in this study.

 For the MDA samples with the lowest amount of template DNA, such as “cells5” and “gDNA8”, there was no correlation to any of the other samples despite the co-localization of several coverage peaks. Even so, when plotting the coverage data between datasets obtained from high and low amounts of template DNA, such as for example from "cells2" and "cells5", we observed that high-coverage regions in sample "cells5" were also over-represented in sample "cells2" (Figure 4). However, some of the highest coverage regions in sample "cells2" contained no reads in sample "cells5", which explains the overall lack of correlation. This indicates that the pattern of the amplification is similar in all samples, but gets increasingly noisy with lower amounts of template DNA, presumably because of stochastic variations in the order in which the primers bind to the few molecules of template DNA available. 

#### GC content and amplification bias

**Figure 5:**
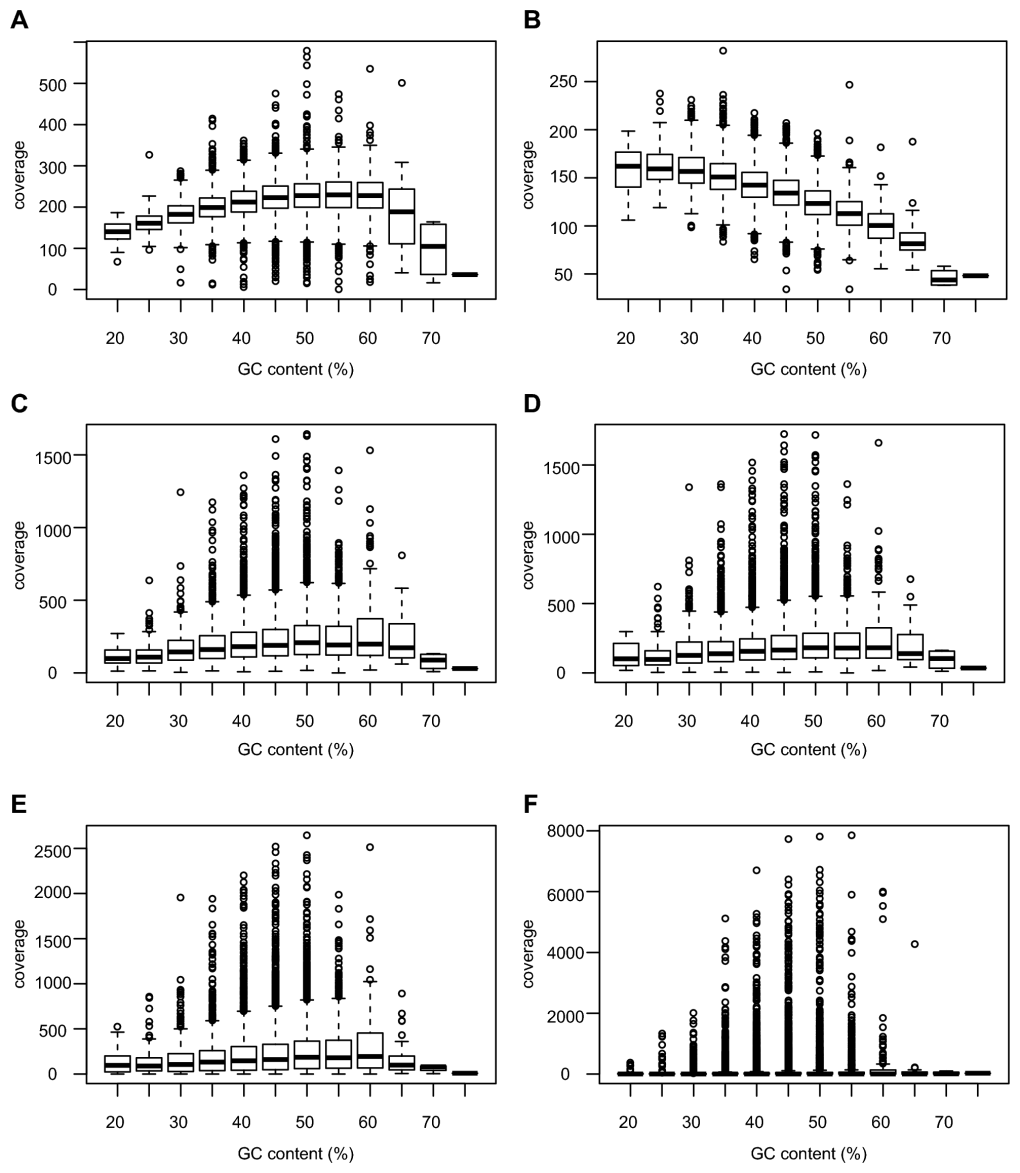
Coverage versus genomic GC-content for MDA samples obtained from bacterial cells. The coverage of sequence reads in relation to the G+C content of the *B. australis* genome is shown for Illumina sequences generated from samples consisting of (**A**-**B**) unamplified and (**C**-**F**) amplified DNA. The unamplified Illumina data sets were obtained from (**A**) the re-sequencing of the *B. australis* genome in this study and (**B**) the previously published *B. australis* genome. The amplified Illumina data sets were obtained from the re-sequencing of the *B. australis* genome from MDA samples taken from a dilution series of intact bacterial cells, as detailed in Table 1. (**C**) "cells2" (**D**) "cells3", (**E**) "cells4" and (**F**) “cells5”. The genomic GC content and sequence reads coverage was averaged over 100 bp windows and sorted into bins that represent 5% intervals in GC content values.

Since GC content is known to influence the efficiency of PCR and sequencing, we investigated whether there was any correlation between the coverage and the local GC content (Figure 5, Figure S2). For all sequence data sets generated as part of this study, including the unamplified control, there was a tendency towards lower coverage over regions with extremely high or low GC content. Furthermore, the Illumina data-set from the *B. australis* genome project had a higher coverage at regions with low GC-content compared to the other samples, demonstrating that sequencing technology and library preparation steps can also influence the coverage. Overall, we could not detect any correlation between the amplification bias and the GC content.

#### Chimeric reads and read pairs

Since the MDA reaction is known to generate chimeric sequences, we investigated whether the proportion of chimeric sequences in the samples correlated with the amount of template used, based on the bitwise flags added to the reads when mapped on the reference genome. We calculated the percentage of putative chimeric read-pairs in each sample as the proportion of non-proper read-pairs, out of all pairs where both reads were mapped (Table 3). Using this method, we identified approximately 3% chimeric read-pairs in both the unamplified control and the MDA samples, indicating that most of the chimeras were formed during the sequencing library preparation step.

**Table 3:** Genome recovery, contamination and chimeric reads from *B. australis* cells and extracted DNA.

Sample^a^	Reads mapped (%)	Genome recovery (%)^b^	Chimeric reads (%)^c^	Chimeric reads mapped on same strand (%)^c^
control	96.1	100	3.0	0.02
cells2	95.2	100	3.3	0.25
cells3	94.9	100	3.3	0.39
cells4	93.6	97.0	2.9	0.45
cells5	81.9	38.4	2.9	0.53
gDNA1	96.0	100	3.0	0.11
gDNA5	94.8	100	3.2	0.34
gDNA6	94.5	100	3.1	0.46
gDNA7	86.9	97.5	3.3	0.55
gDNA8	60.6	45.3	3.6	0.55

^a^ The control sample corresponds to the unamplified sample sequenced in the current study. All other samples correspond MDA samples as detailed in Table 1 and 2.

^b^ The percentage of genome positions with at least one mapped read (see methods)

^c^ The percentage of chimeric reads was calculated as the fraction of all read-pairs where both reads mapped, based on the bit-wise flags added to the reads when mapping to the reference genome (see methods).

 To investigate the putative chimeric read-pairs in more detail, we further categorized the non-proper pairs according to the relative orientation of the mapped reads for each pair. This analysis revealed that although only 0.02% of these read pairs were mapped to the same strand in the control sample, the frequency of such chimeras increased 10-20 fold in the MDA samples (Table 3). This pattern is consistent with the proposed mechanism of chimera formation during MDA [33], where displaced 3'-end strands will most frequently prime on nearby 5'-end strands resulting in inverted sequences.

 In conclusion, while the large majority of chimeric read-pairs in the MDA samples were likely generated during the library preparation step, the frequency of inversions generated by the MDA reaction increased with decreasing amounts of template DNA in the same manner as the magnitude of amplification bias.

### *De novo* genome assembly of MDA data

#### Genome recovery

With a strong bias in coverage when low amounts of template DNA is used in the amplification reaction, a concern for *de novo* genome projects is allelic drop-out, i.e. that parts of the genome are not amplified at all, in which case these genomic regions will not be represented in the sequencing data. If these regions are amplified, but only to a very low level, the sequences can be “rescued” by sequencing in sufficient depth. It is therefore of interest to quantify the fraction of the genome that is represented in each dataset. The sequence datasets produced from the samples with the five highest amounts of template DNA contained reads that covered the complete *B. australis* genome (Table 3). Samples “cells4” and “gDNA7” had reads covering 97% and 98% of the reference genome, indicating that even when the estimated amount of template was only 10-100 cells most of the genome was amplified. For the samples “cells5” and “gDNA8”, which approached single cell levels, only 38% and 45% of the genome was amplified. Although pooling all reads from both of these samples increased the genomic recovery to 65%, there was still 35% of the genome that had no coverage at all. This might however be expected based on the non-random pattern of the amplification bias. 

#### *De novo* genome assembly

The task of genome assembly becomes increasingly more complicated with high levels of amplification bias and chimeric sequences, as many assemblers depend on even coverage across the genome to determine the copy number status of a region, and correct read pair linkage is required to build scaffolds. To investigate the effect of biased amplification and the inclusion of chimeric reads on the genome assembly, we assembled the Illumina data from the MDA samples *de novo* using the SPAdes genome assembler, which was designed specifically for MDA data [34]. For comparison, the assemblies were run in both multi-cell mode (intended for non-MDA data) and single-cell mode (intended for MDA data). We evaluated each assembly in terms of the number of contigs produced, the total length of contaminant contigs and the percentage of the genome represented in the assembly. We also estimated the N50 length, which corresponds to the length of the shortest contig that must be included to reach half the total assembly length after sorting the contigs from longest to shortest and consecutively adding them together. The N50 length is a very common genome assembly statistic and is used as a measure of the level of assembly fragmentation. Surprisingly, the assembler produced very similar results for the single-cell mode and multi-cell mode on the MDA samples (Table 4, Table S3). However, the assembly of the unamplified control sample yielded fewer contigs and a higher N50 value in the multi-cell mode.

**Table 4:** Spades De novo assembly statistics, single-cell mode^a^.

Sample^b^	Nb. contigs > 500bp	Aligned contigs	Unaligned bases (bp)^c^	N50 of aligned contigs (bp)	Genome recovery (%)^d^
control	55	54	690	153519	99.96
cells2	62	58	6365	172425	99.94
cells3	67	56	12095	141851	99.97
cells4	128	111	27933	70292	95.62
cells5	198	80	184234	45487	35.08
gDNA1	52	52	0	172505	99.94
gDNA5	79	59	19799	172490	99.93
gDNA6	82	53	42039	200568	99.98
gDNA7	238	127	182944	52846	96.68
gDNA8	330	54	389894	27118	30.86

^a^ MDA samples were assembled *de novo*, and the resulting contigs were aligned to the *B. australis* reference genome sequence

^b^ The control sample correspond to the unamplified sample sequenced in the current study. All other samples correspond to MDA samples as detailed in Table 1 and 2.

^c^ The total length of contigs which did not align to the *B. australis* reference sequence

^d^ The percentage of genome positions covered by at least one assembled contig

**Figure 6:**
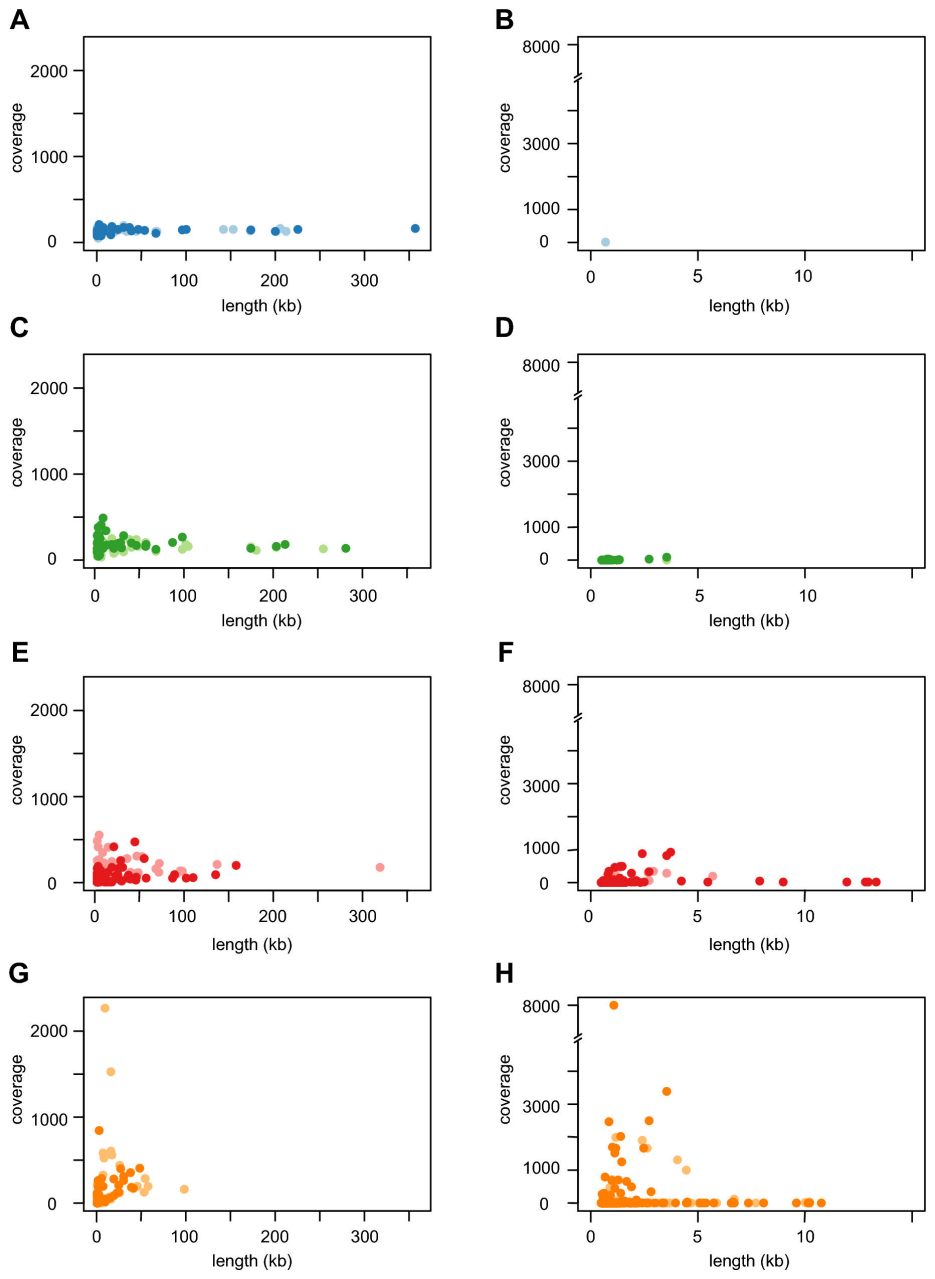
Coverage and length of contigs obtained from *de* *novo* assembly. The plots show the coverage and length of contigs (> 500 bp) obtained from the assembly of sequence reads after the re-sequencing of the *B. australis* genome from MDA samples. The contigs have been separated into those that show significant BLAST hits to the *B. australis* genome in the left column (**A**, **C**, **E**, **G**) and those that do not in the right column (**B**, **D**, **F**, **H**). (**A**-**B**) Dark blue refers to the unamplified Illumina data set obtained from the re-sequencing of the *B. australis* genome in this study. All other colors refer to the Illumina data sets obtained after re-sequencing of the *B. australis* genome from MDA samples where dilution series of intact bacterial cells and genomic DNA were used as template, as detailed in Tables 1 and 2, respectively. (**A**-**B**) "gDNA1" (light blue), (**C**-**D**) "cells2" (light green) and "gDNA5" (dark green), (**E**-**F**) "cells4" (light red) and "gDNA7" (dark red), (**G**-**H**) "cells5" (light yellow) and "gDNA8" (dark yellow).

 Overall, most regions in the genome that contained mapped reads were also represented in the contigs from the *de novo* assembly (Tables 3 vs Table 4). However, the assemblies became increasingly fragmented with decreasing amounts of the template DNA (Table 4, Figure 6 (left column)). For example, 62 contigs larger than 500 bp was obtained for sample “cells2” (of which 58 aligned to the reference genome), while 128 contigs were assembled for sample “cells4” (of which 111 aligned to the reference genome). Similarly, the N50 value of the aligned contigs decreased from 172 kb in the sample “cells2” to 45 kb in the sample “cells4”. Differences between the assembled contigs and the reference genome, as identified by analysis with both MUMmer and Artemis comparison tool, were small and generally occurred at the ends of the assembled contigs. Thus, the main effect of the amplification bias and the inverted read-pairs was a reduction in the contig sizes, rather than mis-assembled contigs.

#### Contamination

We suspected that samples with a low fraction of reads that mapped to the reference genome contained contaminating DNA. The presence of amplified contaminating DNA is particularly problematic if the contaminating sequences assemble into contigs with a similar size and coverage as the contigs of the genome that was the intended target for the sequencing. We therefore investigated the contigs from the SPAdes assemblies that were longer than 500 bp and did not align with the reference *B. australis* genome. The contaminating contigs were mostly short and of low coverage (Figure 6, (right column)). However, as the amount of template DNA decreased, the coverage and size of the contaminating contigs increased. At the lowest template concentrations (“cells5” and gDNA8”), some contaminating contigs had more than a thousand fold coverage and others were up to 10 kb long. Further complicating the situation was that several of the expected *B. australis* contigs in these samples were short and had low coverage. A blast search with the contaminant contigs against the nt database indicated several different sources of contamination and a large number of contigs with no hits. For the contigs that had highly significant hits, a large proportion appeared to be derived from various vector and phage sequences. Several contigs matched the bacterium *Delftia acidovorans*, which has previously been identified as a contaminant in DNA samples amplified with the MDA method [16,21,31], and which was also identified in a subset of MDA samples prior to Illumina sequencing (Table 1).

### Amplification bias in endosymbiont genomes

To investigate the generality of the results obtained with the *B. australis* test data set, we re-analyzed sequence data from two published genome projects on uncultured obligate endosymbionts, *Wolbachia* [17] and *M. mitochondrii* [14,35] (Table S2). In both studies, the MDA reaction was applied to samples consisting of intact bacterial cells, but different protocols were used to isolate the bacterial cells from the host cells. The *Wolbachia* cells were isolated by a series of differential centrifugation and filtration steps, and the bacterial pellet was used directly for amplification with the REPLI-g Midi kit. The *M. mitochondrii* cells were isolated from dissected ovaries using microcapillaries, and the MDA was applied directly to the cytoplasmic preparations using the REPLI-g Mini kit. Thus, in both studies, an unknown number of bacterial cells were used as template for the MDA reaction.

 As in the case of the amplified DNA of *B. australis*, a non-random amplification pattern was previously noted in the samples from *Wolbachia* [17] (Figure S3A-B). The correlation coefficients between the coverage in the *Wolbachia* samples were in the range of the highest values for the *B. australis* samples (0.61-0.75), suggesting that relatively large quantities of cells were used as templates in these reactions. Indeed, all datasets from the *Wolbachia w*Ha genome showed cumulative coverage distributions equal to or less biased than sample “cells2”(Figure S4A). Similarly, for *Wolbachia w*No, both the single-end and paired-end 454 data showed less bias than sample “cells2”, but the Illumina data-set indicated a level of bias somewhere between samples “cells3” and “cells4” (Figure S4B). 

Likewise, a non-random pattern of amplification was observed for the *M. mitochondrii* dataset (Figure S3C). The paired-end data for *M. mitochondrii* had a level of bias close to sample “cells4”, which is relatively higher than the *Wolbachia* samples and the other *M. mitochondrii* samples, indicating that the amount of template DNA in this sample was particularly low (Figure S4C). Indeed, the correlation coefficients for the *M. mitochondrii* data were somewhat lower than for the *Wolbachia* data in all comparisons (0.41-0.47). We conclude that both the magnitude and the pattern of the amplification bias in the data sets obtained from *Wolbachia* and *M. mitochondrii* were comparable to the results obtained from *B. australis* when using relatively high quantities of template DNA in the amplification reaction.

Similarly to the *B. australis* samples, we found no correlation between coverage and GC content in the *Wolbachia* and *M. mitochondrii* data sets (Figures S5 and S6). The regions with the highest coverage in *Wolbachia* tended to have a slightly lower GC content compared to the corresponding regions in *B. australis*, consistent with a lower mean genomic GC content (34-35% for *Wolbachia* compared to 42% for *B. australis*). These results lend additional support to the notion that variations in GC content are not the cause of the amplification bias*.*

For the previously published short-insert libraries and single-end reads (330-400 bp), the overall percentages of chimeras ranged from 1-4% (Table S4), similarly to 3-4% obtained for paired end reads from short-insert libraries (250 bp) in this study (Tables 3 and 4). In contrast, for long-insert libraries (2300-2700 bp), the percentage of read pairs mapped as "false" by Newbler ranged from 13-33%. The majority of these pairs were mapped in the same orientation, indicating that they were formed during the MDA reaction. While the percentage of pairs mapped on the same strand was 9% in the two independent MDA samples from *Wolbachia*, it was as much as 23% for *M. midichloria*. Given that the long-insert library sample of *M. midichloria* also had a higher level of amplification bias compared to the other samples, it seems likely that this difference could be explained by a difference in the amount of template. However, since no unamplified control sample is available for long-insert libraries it is not possible to infer how much the MDA reaction *per se* contributed to the formation of these chimeric read pairs.

Between 94-99% of the reads obtained from all *Wolbachia* MDA samples mapped back to their reference genomes (Table S4). Similarly, 99% of the *M. mitochondrii* MDA samples sequenced with single-end 454 sequencing technology mapped to the reference genome (Table S4). These levels are comparable to those obtained in the re-sequencing of the *B. australis* genomes when using the highest concentrations of template DNA in the amplification reactions (Table 3). In contrast, only 67.7% of the reads from the *M. mitochondrii* MDA sample obtained with paired-end 454 sequencing technology mapped back to the reference genome. A *de novo* assembly of the 33% unmapped reads yielded a short contig with similarity to the typical MDA contaminant *D. acidovorans*, and another short contig with similarity to the tick host. Most of the reads (20.089 reads) were in a single contig of 2.6 kb which had weak hits to various plasmids and vectors. Artificial sequences, possibly stemming from short pieces of contaminating DNA of plasmids or vectors present the amplification kit or the environment, could potentially be generated by the MDA reaction when template DNA is limited and there is little DNA from contaminating bacteria. 

## Discussion

### Non-random amplification bias in MDA samples

We have shown in this study that previously identified problems associated with the MDA reaction, such as amplification bias, generation of chimeric reads and amplification of contaminant DNA, are strongly dependent on the amount of template DNA used. The lower the amount of DNA in the sample, the stronger the amplification bias, as has also previously been observed using comparative microarray hybridization on MDA samples [36], and more recently by Illumina sequencing of MDA samples [12]. Both the current dataset and our reinvestigation of previously sequenced endosymbiont genomes confirmed that independent amplifications of the same genome with MDA produce similar patterns in read coverage. This demonstrates that the amplification pattern is highly reproducible for each genome. 

 These findings are remarkable since the amplification bias is generally assumed to be random [4,26-29]. One explanation for the contradictory results might be that most of the previously published studies have amplified DNA from single cells. Non-random trends of amplification during the MDA reaction have been observed before, but in experiments in which the amount of template DNA ranged from 1ng to 1μg, which is much higher than what was used in most of our samples. Furthermore, these earlier studies did not evaluate the amplification bias based on genome sequence data, but on data from microarrays [10,37], qPCR [11], small shot-gun sequencing libraries [8] and rRNA gene amplicon pyrosequencing [38]. As we have demonstrated here for whole-genome sequence data, the amplification profile was highly reproducible for samples containing more than 100 cells or 10^-4^ ng of genomic DNA, but became increasingly random when the quantity of template DNA approached that of single cells. 

 The amplification bias is presumably generated early in the MDA reaction, during the initial rounds of priming and amplification of the template DNA, and the high reproducibility of the pattern thus indicates that the initial binding of primers might not be random. However, when priming occurs on a single or a few molecules of template DNA, it is likely that stochastic variations in the binding of primers might result in fewer and higher coverage peaks than when hundreds or thousands of molecules are used to start the reaction, resulting in a less reproducible pattern for the lowest quantities of template DNA. 

 One hypothesis is that the amplification bias is determined by the composition of primers in the commercial kits. If so, a non-random amplification pattern could be due to uneven aliquots of the random primers. However, since we observed a non-random bias in three different data sets produced at three different time points in two different laboratories, we believe that the bias is not simply due to one specific batch of the Repli-G kit that was used for the amplifications of the *B. australis* DNA. A non-random amplification bias has also been observed for other kits, such as Genomiphi that was used to amplify *Chlamydia* cells from clinical samples [12]. This does not necessarily mean that all kits have the same performance characteristics. Wang et al. [37] found correlations between the coverage profiles for samples amplified with the same kit, but not for samples amplified with different kits. Yilmaz et al. [38] found differences in the quantitative representation of bacterial strains in amplifications from mixed samples that were related to the kit used for the amplification. Thus, although differences between kits have been noted, the non-random amplification pattern seems to be a general phenomenon.

 The amplification bias could potentially also be influenced by the state of the chromosome, gene expression, GC-content or other nucleotide composition traits. Indeed, Yilmaz et al. [38] found that GC-rich bacterial genomes in mixed samples were less well amplified than other genomes with two of the three kits tested. No such simple correlation between the bias and the variation in GC content across the genome was observed for any of the datasets that we investigated. Regions with extremely high or low GC-content were in all data sets poorly covered by sequence reads, even in the non-amplified genome sequences, suggesting that this effect is rather due to the PCR amplification step during library construction for the Illumina sequencing [39]. 

 If the amplification profile can be modified by the primer mix this could potentially be used to improve the MDA method. Currently, *de novo* genome assemblies of sequences from single-cell amplified genomes are designed by pooling MDA samples from several individual cells. Thus, pooling MDA samples amplified with different primer sets might lead to a more even coverage of the genome. For MDA samples on multiple cells, e.g. endosymbiont populations, the benefit of such an approach would have to be balanced against a higher overall level of the amplification bias due to splitting the template sample into smaller cell numbers. 

### Advantages of using multiple cells or genomes as templates for MDA

As seen in this study, even when using only a few hundred or a few thousand bacterial cells as the template most of the genome was successfully amplified and most of the artifacts associated with MDA became negligible. This suggests that the MDA reaction is particularly useful for genome amplification when the amount of template cells is low but exceeds a single cell. Below, we discuss the impact of the quantity of the template DNA on three such artifacts.

First, the magnitude of the amplification bias was much reduced when a larger amount of template material was used. In projects where the goal is to generate a complete genome by *de novo* assembly, even a small reduction in the magnitude of the amplification bias is valuable. Although several new assemblers have been developed recently specifically to handle MDA data with uneven coverage [34,40,41], genome assembly on such data remains challenging. A striking result was that the same assembler generated more complete assemblies when the genome had been amplified from larger amounts of template material. Notably, the improvements in the assembly were particularly large in comparisons to samples with the lowest amounts of template DNA, indicating that even a small increase in template DNA has a strong impact on the assembly.

Second, the formation of chimeric reads and read pairs was also correlated with the amount of template material used in the reaction. Thus, the Illumina sequences from *B. australis* displayed a small but consistent increase in the percentage of inverted read pairs with decreasing quantities of template DNA. Both insert sizes and sequencing library preparation methods may potentially influence the number of chimeric reads [42]. In our study, the most striking effect was related to the insert size. For example, the relative fraction of inverted read pairs increased from 1-2% to 10-20% as the insert sizes increased from 250-400 bp to 2,000-2,500 bp. Consistently, Lasken et al. [33] found that the large majority of chimeras formed during the MDA reaction were inversions. The library preparation steps seemed to have a much smaller influence on the generation of chimeras. The number of chimeras in single-end 454 and paired-end Illumina data with read lengths of 350-400 bp were very similar (1-4%), and hence we do not believe that the library methods differ in this respect. Unfortunately, we had no unamplified control for the *Wolbachia* read pairs generated with the 454 technologies and therefore we do not know how many of the chimeras were contributed by the library preparation step for these samples. However, the overall fraction of chimeric read pairs (33%) for the 454 data with 2 kb inserts from *M. mitochondrii* was identical to the fraction of chimeric read pairs (33%) for 3 kb insert-size libraries from *Escherichia coli* amplified from single genomes and sequenced with the Sanger sequencing method [28]. This suggests that the quantity of template DNA used in the amplifications of *M. mitochondrii* was particularly low. We conclude that most of these chimeras were generated during the MDA reaction itself and that the fraction of chimeric reads and read pairs can decrease up to an order of magnitude as the amount of template DNA increases. 

In general, large-insert paired-end libraries have been discouraged for MDA projects, because the prevalence of chimeric read pairs increases with the distance between the reads [43]. Even so, long insert libraries can be useful in the assembly process, as demonstrated by the assembly statistics from the two *Wolbachia* genomes. When adding the paired-end 454 datasets to the *de novo* assemblies made from the single-end 454 reads for the two *Wolbachia* strains, the 300-400 contigs could be merged into only 1-3 scaffolds. The suggested scaffolds were confirmed by PCR on unamplified DNA, confirming that the presence of chimeric read-pairs did not disrupt the correct assembly in this case. However, the results of the current study also showed that the utility of long-insert size libraries is likely to depend on the amount of template cells used in the MDA reaction. Long insert size libraries should therefore only be constructed if quantities in the order of a few thousands cells are used as templates for the MDA reaction.

 A third advantage of using multiple cells as templates for the MDA reaction is a lower sensitivity to contamination. Many solutions have been proposed to reduce the impact of contamination [27,28,31,44] but in general these approaches are work-intensive and/or expensive. For *de novo* genome projects, the distinction between target contigs and contaminant contigs becomes increasingly difficult to make when the contaminating contigs reach a length and coverage similar to the target contigs, as was observed for the MDA samples with the lowest amount of template. Furthermore, assemblies of MDA samples from extracted genomic DNA contained more contigs from contaminating DNA compared to the assemblies of MDA samples made from cells. This suggests that cells are preferable as templates for the MDA reaction, perhaps because the extra step of DNA extraction produces more opportunities for contaminating DNA to be introduced. It should be recalled that the experiments reported here were performed in a standard laboratory without specialized equipment to control for contamination and using a commercial whole-genome amplification kit, yet only samples generated from the lowest amounts of template material were significantly affected by contamination.

For all of these reasons, it is desirable to increase the number of template cells. An alternative approach is to increase the number of genome copies per cell. One such recently developed method was to artificially induce polyploidy in *Bacillus subtilis* by inhibiting the bacterial cytoskeleton protein FtsZ [45]. A two to four-fold increase in genome copy numbers resulted in less amplification bias and higher genome recovery [45], consistent with our findings that the efficiency of the MDA reaction was improved even for slight increases in the amount of template DNA. Interestingly, insect endosymbionts such as *Buchnera aphidicola* [46,47], *Blochmannia floridanus* [48] and *Blattabacterium* [49] are natural polyploids with hundreds of genome copies per cell. As such, these genomes should be particularly suitable for amplification with the MDA method. Indeed, the 240 kb genome of Candidatus Sulcia *muelleri*, which is naturally polyploid, was successfully amplified from a single bacterial cell and assembled into a closed genome [16]. As yet we do not know what changes in the endosymbiont genomes have resulted in polyploidy, but it is intriguing to speculate that knowledge about these processes might provide solutions to some of the major challenges in single cell genomics.

Given that most bacteria cannot be cultured, it is likely that MDA will be used more widely in the future, as we are awaiting new sequencing technologies and library preparation methods that are capable of handling ultralow quantities of DNA. Currently, the lowest quantity of DNA which can be used directly for the generation of a next-generation sequencing library is approximately 1 ng [50]. However, the method of transposase-mediated fragmentation of DNA also produces an uneven sequence representation which is dependent on the nucleotide composition of the template DNA [50]. The MDA reaction is particularly useful for amplification of DNA from highly clonal bacterial populations, such as obligate endosymbionts of insects with multiple genome copies per cell and multiple bacterial cells per insect. Prior to the invention of MDA, thousands of insects were needed for the extraction of enough bacterial DNA for sequencing. Amplifying the DNA from a few bacterial cells of high purity may be preferable for such samples, as the collection of large amounts of material will increase the risk of sampling non-clonal variants [51]. With the aid of the MDA method it is in principle possible to identify and trace the spread of vector-born infectious diseases through sampling, amplification and sequencing of bacterial genomes from single insects. As we have shown here, such studies can now be performed almost as efficiently as direct sequencing of genomes of cultivated bacteria.

## Materials and Methods

### Cultivation and amplification of *B. australis*

*B. australis* cells were cultured on blood-agar plates for 13 days, to a mat-like density. Cells corresponding roughly to one colony were collected into a tube with 500µl PBS and diluted in 10x increments with PBS. MDA was applied to all dilutions using 3 µl of each dilution as template with REPLI-g Midi kit (Qiagen), according to the manufacturers instructions (cell protocol). Additionally, the amount of cells in each of the dilutions was estimated from a plate colony count using the remaining cell solution.

*B. australis* DNA was extracted from bacterial cells grown on blood-agar plates using the “AquaPure Genomic DNA kit” (BioRad) with protocol 3.6 (gram-negative DNA), and including an RNase treatment. The concentration of the extracted DNA was measured spectrophotometrically on a NanoDrop instrument (Thermo Scientific) and diluted to contain an estimated 34 ng of DNA. This template solution was then further diluted in 10x increments with TE buffer. MDA was carried out on all dilutions using 2.5µl of each dilution as template with REPLI-g Midi kit (Qiagen), according to the manufacturers instructions (genomic DNA protocol).

### Sample selection and sequencing of *B. australis*

To estimate the genomic recovery of *B. australis* in each MDA sample and select samples for Illumina sequencing, an aliquot of each of the MDA products was diluted and used as template for PCR. Three sets of MLST primers were used (16S, *batR, gltA*) [52], which are approximately equidistant on the circular genome.

The MDA samples representing the four lowest template concentrations containing amplified *B. australis* DNA (as estimated from PCR) from both the cell and DNA dilution series were selected for re-sequencing. Furthermore, the MDA sample generated from the highest concentration of DNA template (34 ng) was sequenced, since this quantity is within the concentration range recommended by the manufacturer. Additionally, the undiluted DNA sample from *B. australis* (i.e. unamplified DNA), which was the starting material for the serial dilution samples that were used as template for the MDA reactions on genomic DNA, was included as a control.

The MDA samples were purified with QiaAmp DNA mini Kit (Qiagen) according to the manufacturers instructions. All 10 *B. australis* samples were sequenced at the same time in one flow-cell on an Illumina MiSeq instrument, generating 2 x 150 bp reads from standard MID-tagged paired-end libraries with insert sizes around 250 bp. 

### Bioinformatic analysis

The Illumina sequence data was filtered for low-quality reads with Trimmomatic [53]. The minimum length of reads to be kept after filtering was adjusted to the read length in the dataset, and was set to 36 bp for 38 bp reads, 95 bp for 100 bp reads, and 130 bp for 150 bp reads. All other filtering settings were identical for all Illumina datasets. Filtered reads were mapped to their corresponding reference genome sequences using bwa [54] with default settings. The output sam-files were converted to bam-format, sorted on coordinates and marked for duplicates using Picard tools (http://picard.sourceforge.net). 454 reads were mapped with the gsMapper (454 Life Sciences Corp., Roche, Branford, CT 06405, US) without pre-filtering, and the resulting sam-file was treated the same as for the Illumina data. 

Coverage was extracted from the duplicate-flagged bam-files using the depth command in samtools, which measures the number of reads covering each base in the genome, excluding reads flagged as duplicates. To evaluate the percentage of the genome represented in each dataset, all single positions with no reads mapping to them were counted as missing, while all positions with at least one read mapping were counted as present.

All figures were made with R (R development core team 2011). For coverage plots and cumulative coverage distributions, the coverage was first averaged in 100 bp windows. The datasets were scaled to have the same mean as the control sample for *B. australis* samples, or the first sample plotted, for *Wolbachia* and *M. mitochondrii* data. Coverage plots were generated by applying a moving average filter with two window sizes (300 bp and 500 bp) on the scaled data, using the package "signal". 

To evaluate the correlation between amplification bias in independent MDA reactions, the coverage was averaged in 1000 bp windows, and the data was scaled to have the same mean coverage. Pairwise values of Kendalls rank correlation coefficients were calculated in R and plotted using the "heatmap.2" function in the package "gplots" and the package "RColorBrewer". To compare GC content and coverage, the GC content was averaged in 100 bp windows along the genome and paired with the average coverage of the corresponding windows. The paired data was sorted according to GC content in bin-sizes of 5% and plotted in R. 

The percentage of chimeric reads in the Illumina datasets was estimated based on the bitwise flags added by the bwa mapper in the bam-files using samtools [55]. Starting with all read-pairs for which both reads were mapped, the reads flagged as "proper" were inferred to be correct, and read-pairs not flagged as "proper" were inferred to be putative chimeras. The putative chimeras were further categorized according to whether the reads in a pair mapped to the same strand or to different strands, based on additional flags. For the 454 single-end data, the percentage of chimeric reads was taken directly from the “454NewblerMetrics.txt” output file. For the 454 paired-end reads, the percentages were extracted from the Newbler output file “454PairStatus.txt”, where "false" pairs were inferred to be putative chimeras. The "false" pairs were further categorized, based on whether they were mapped in the same orientation, facing outwards or too distant from each other, which is also specified in the "454PairStatus.txt" file.

*De novo* genome assemblies of *B. australis* samples were made with the SPAdes genome assembler [34], which was run in both single-cell mode and multi-cell mode on all samples. The assemblies were evaluated with MUMmer [56], using the perl script "dnadiff" and excluding contigs smaller than 500bp, and visually inspected using Artemis comparison tool [57] Unaligned contigs were analyzed by running BLAST [58] against the nt database at NCBI. Blast hits to Enterophage phiX were manually removed, as DNA from this sequence is used as a control by the sequencing platform. Blast hits were considered significant, if E < e^-05^, the percentage identity above 95% and the alignment to the hit was at least 80% of the query length.

#### Accession Numbers

The Illumina data from the *B. australis* genome obtained in this study from the samples “cells2-cells5” and “gDNA1, gDNA5-gDNA8” as well as the Illumina data from the published *B. australis* genome sequence [28] have been deposited in the Genbank Sequence Read Archive under BioProject ID PRJNA222516.

## Supporting Information

Figure S1 **Concentration-dependent bias in read coverage of MDA samples obtained from genomic DNA.** The coverage of sequence reads across the *B. australis* genome is shown for Illumina sequences generated from (**A**) unamplified DNA in the previously published *B. australis* genome project and (**B**-**F**) re-sequencing of the *B. australis* genome from MDA samples. Five different dilutions of genomic DNA were used as templates for the MDA reaction (B) "gDNA1", (**C**) " gDNA5", (**D**) " gDNA6" (**E**) “gDNA7” and (**F**) " gDNA8", as detailed in Table 2. For each plot, the coverage of the unamplified control obtained from the re-sequencing of *B. australis* in this study is shown in red, and the sample for comparison in blue (two shades, corresponding to two window sizes). The mean coverage of all samples was scaled to be the same as the control sample.
(TIF)

Figure S2 **Coverage versus genomic GC-content for MDA samples obtained from bacterial cells.** The coverage of sequence reads in relation to the G+C content of the *B. australis* genome is shown for Illumina sequences generated from samples consisting of (**A**) unamplified and (**B**-**F**) amplified DNA. The unamplified Illumina data set was obtained from the re-sequencing of the *B. australis* genome in this study. The amplified Illumina data sets were obtained from the re-sequencing of the *B. australis* genome from MDA samples where a dilution series of genomic DNA was used as template, as detailed in Table 2. (**B**) "gDNA1" (**C**) "gDNA5", (**D**) "gDNA6", (**E**) “gDNA7” and (**F**) “gDNA8”. The genomic GC content and sequence reads coverage was averaged over 100 bp windows and sorted into subsets that represent 5% intervals in GC content values.
(EPS)

Figure S3 **Non-random patterns in read coverage for endosymbiont genomes.** The coverage of sequence reads across the genomes of (**A**) *Wolbachia* strain wHa, (**B**) *Wolbachia* strain wNo and (**C**) *M. mitochondrii* for three independent MDA reactions. (**A**) MDA on *Wolbachia* strain wHa: brown = single-end 454, blue = paired-end 454, green = paired-end Illumina. (**B**) MDA on *Wolbachia* strain wNo: brown = single-end 454, blue = paired-end 454, green = paired-end Illumina. (**C**) MDA on *M. mitochondrii*: brown = single-end 454 (GS-FLX), blue = paired-end 454, green = single end 454 (Titanium). For all data sets, the coverage of the samples plotted in green and blue were scaled to have the same mean coverage as the sample plotted in brown.
(EPS)

Figure S4 **Cumulative read coverage distributions of the MDA samples.** The graph displays the relative fraction of 100 bp windows with a mean coverage below or equal to the coverage given on the x-axis. The sample "control" refers to the unamplified Illumina data set obtained from the re-sequencing of the *B. australis* in this study (grey). In green-blue shades, the figures contain the following: (**A**) *w*Ha,1 : paired-end 454 data, *w*Ha,2: single-end 454 data, *w*Ha,3: Illumina data. (**B**) *w*No,1: paired-end 454 data, *w*No,2: single-end 454 data, *w*No,3: Illumina data. (**C**) midi.1: paired-end 454 data, midi.2: single-end 454 data (GS-FLX), midi.3: single-end 454 data (Titanium).
All plots contain the MDA samples “cells2-5” (orange-red shades) for comparison and all datasets were scaled to have the same mean coverage as the sample "control".
(TIF)

Figure S5 **Coverage versus genomic GC-content for MDA samples obtained from *Wolbachia* cells.** The coverage of sequence reads in relation to the G+C content of the *Wolbachia* genome is shown for two strains and three independent MDA reactions. (**A**) Sample "*w*Ha, single-end", (**B**) Sample "*w*No, single-end", (**C**) Sample "*w*Ha, paired-end", (**D**) Sample "*w*No, single-end", (**E**) Sample "*w*Ha, Illumina", (**F**) Sample "*w*No, Illumina". The genomic GC content and sequence reads coverage was averaged over 100 bp windows and sorted into bins that represent 5% intervals in GC content values.
(EPS)

Figure S6 **Coverage versus genomic GC-content for MDA samples obtained from *M. mitochondrii*.** The coverage of sequence reads in relation to the G+C content of the *M. mitochondrii* genome is shown for three independent MDA reactions. (**A**) Sample "454 (GS-FLX)", (**B**) "paired-end 454" and (**C**) "single-end 454 (titanium)". The genomic GC content and sequence reads coverage was averaged over 100 bp windows and sorted into bins that represent 5% intervals in GC content values.
(EPS)

Table S1 **Sequencing data and quality, *B. australis* samples.** The data was filtered using Trimmomatic as described in the methods section.
(DOCX)

Table S2 **Sequencing data and quality of published sequencing data.** Only Illumina data was filtered prior to mapping.
(DOCX)

Table S3 **Spades De novo assembly statistics, multi-cell mode.**
(DOCX)

Table S4 **Genome recovery, contamination and chimeric reads, published data.**
(DOCX)
