# Supplementary figures and images for: Testing the Reproducibility of Multiple Displacement Amplification on Genomes of Clonal Endosymbiont Populations
Source: PLoS One. 2013 Nov 27;8(11):e82319. doi: 10.1371/journal.pone.0082319 (PMC3842359; doi:10.1371/journal.pone.0082319)

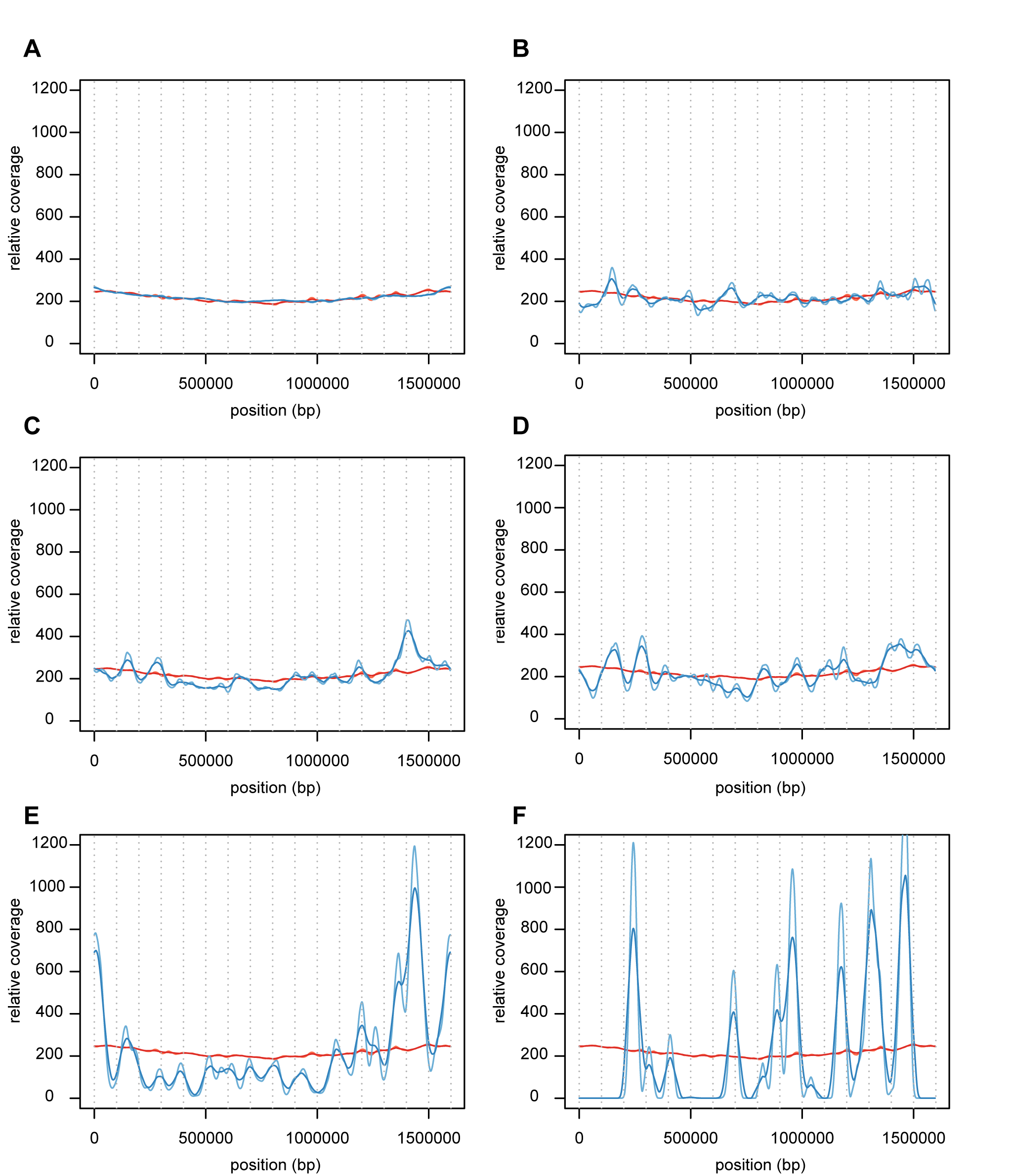

Supplement: Figure S1 — Concentration-dependent bias in read coverage of MDA samples obtained from genomic DNA. The coverage of sequence reads across the B. australis genome is shown for Illumina sequences generated from (A) unamplified DNA in the previously published B. australis genome project and (B-F) re-sequencing of the B. australis genome from MDA samples. Five different dilutions of genomic DNA were used as templates for the MDA reaction (B) "gDNA1", (C) " gDNA5", (D) " gDNA6" (E) “gDNA7” and (F) " gDNA8", as detailed in Table 2. For each plot, the coverage of the unamplified control obtained from the re-sequencing of B. australis in this study is shown in red, and the sample for comparison in blue (two shades, corresponding to two window sizes). The mean coverage of all samples was scaled to be the same as the control sample. (TIF) [file pone.0082319.s001.tif]

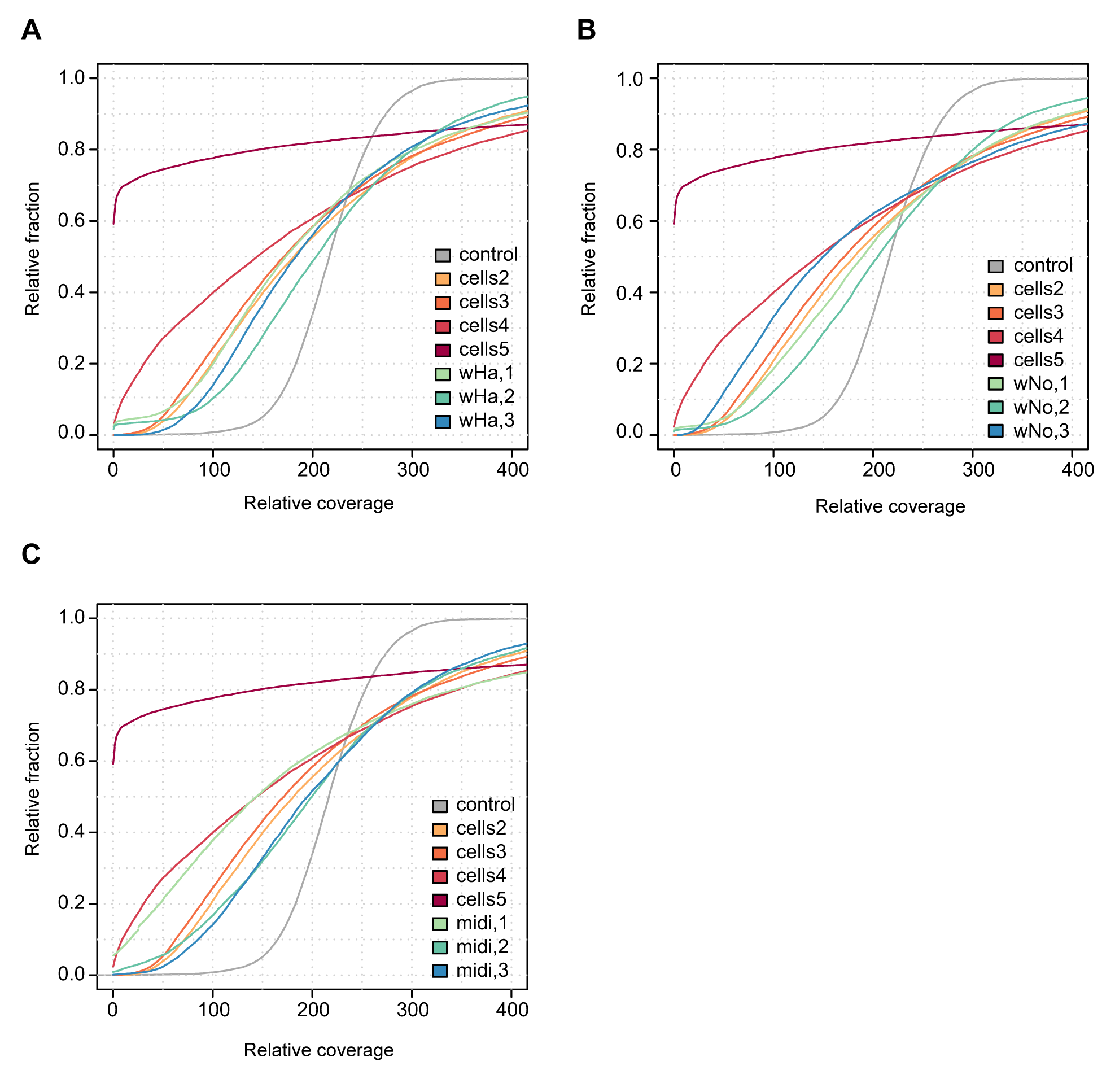

Supplement: Figure S4 — Cumulative read coverage distributions of the MDA samples. The graph displays the relative fraction of 100 bp windows with a mean coverage below or equal to the coverage given on the x-axis. The sample "control" refers to the unamplified Illumina data set obtained from the re-sequencing of the B. australis in this study (grey). In green-blue shades, the figures contain the following: (A) wHa,1 : paired-end 454 data, wHa,2: single-end 454 data, wHa,3: Illumina data. (B) wNo,1: paired-end 454 data, wNo,2: single-end 454 data, wNo,3: Illumina data. (C) midi.1: paired-end 454 data, midi.2: single-end 454 data (GS-FLX), midi.3: single-end 454 data (Titanium). All plots contain the MDA samples “cells2-5” (orange-red shades) for comparison and all datasets were scaled to have the same mean coverage as the sample "control". (TIF) [file pone.0082319.s004.tif]
