# Supplementary material for: Testing the Reproducibility of Multiple Displacement Amplification on Genomes of Clonal Endosymbiont Populations
Source: PLoS One. 2013 Nov 27;8(11):e82319. doi: 10.1371/journal.pone.0082319 (PMC3842359; doi:10.1371/journal.pone.0082319)
Supplement: Table S1 — Sequencing data and quality, B. australis samples. The data was filtered using Trimmomatic as described in the methods section. (DOCX) [file pone.0082319.s007.docx]

**Table S1. Sequencing data and quality, *B.australis* samples**

| Sample^a^ | Raw nb. of read pairs | Nb. read pairs after filtering | Percentage reads passing filtering | Coverage (mean) | CV(std/mean) |
| --- | --- | --- | --- | --- | --- |
| control | 1382164 | 1223392 | 88.5 | 218x | 0.21 |
| cells2 | 1484311 | 1271177 | 85.6 | 222x | 0.71 |
| cells3 | 1326906 | 1178736 | 88.8 | 205x | 0.80 |
| cells4 | 1497164 | 1367861 | 91.4 | 234x | 1.15 |
| cells5 | 1086906 | 959789 | 88.3 | 141x | 3.56 |
| gDNA1 | 1392912 | 1280496 | 91.9 | 227x | 0.41 |
| gDNA5 | 1670261 | 1521661 | 91.1 | 265x | 0.47 |
| gDNA6 | 1463013 | 1328518 | 90.8 | 230x | 0.54 |
| gDNA7 | 1113705 | 968022 | 86.9 | 154x | 1.29 |
| gDNA8 | 1091700 | 893506 | 81.8 | 98x | 2.38 |

^a^ The control sample corresponds to the unamplified sample sequenced in the current study. All other samples correspond MDA samples as detailed in Table 1 and 2.
