# Supplementary material for: Testing the Reproducibility of Multiple Displacement Amplification on Genomes of Clonal Endosymbiont Populations
Source: PLoS One. 2013 Nov 27;8(11):e82319. doi: 10.1371/journal.pone.0082319 (PMC3842359; doi:10.1371/journal.pone.0082319)
Supplement: Table S2 — Sequencing data and quality of published sequencing data. Only Illumina data was filtered prior to mapping. (DOCX) [file pone.0082319.s008.docx]

**Table S2. Sequencing data and quality, published data**

| Sample | Sequencing method | Raw nb. of read/read pairs | Nb. read/read pairs after filtering | Percentage reads passing filtering | Coverage (mean) | CV(std/mean) |
| --- | --- | --- | --- | --- | --- | --- |
| *B. australis* [[1](#_ENREF_1)] | Illumina, paired-end | 3709344 | 3200542 | 86.3 | 136x | 0.22 |
| *Wolbachia, w*No [[2](#_ENREF_2)] | Illumina, paired-end | 8863432 | 6625773 | 74.8 | 876x | 0.98 |
| *Wolbachia, w*No | 454 paired-end (3kb insert) | 663271 | NA | NA | 103x | 0.66 |
| *Wolbachia, w*No | 454 single-end | 793407 | NA | NA | 145x | 0.50 |
| *Wolbachia, w*Ha [[2](#_ENREF_2)] | Illumina, paired-end | 7148563 | 4920135 | 68.8 | 695x | 0.67 |
| *Wolbachia, w*Ha | 454 paired-end (3kb insert) | 702509 | NA | NA | 103x | 0.82 |
| *Wolbachia, w*Ha | 454 single-end | 764450 | NA | NA | 149x | 0.51 |
| *M.mitochondrii*^c^ | 454 single-end (GS-FLX) | 280622 | NA | NA | 43x | 0.59 |
| *M.mitochondrii* [[3](#_ENREF_3)] | 454 single-end (Titanium) | 544117 | NA | NA | 125x | 0.55 |
| *M.mitochondrii* | 454 paired-end | 100574 | NA | NA | 9x | 1.10 |
