# Supplementary material for: Testing the Reproducibility of Multiple Displacement Amplification on Genomes of Clonal Endosymbiont Populations
Source: PLoS One. 2013 Nov 27;8(11):e82319. doi: 10.1371/journal.pone.0082319 (PMC3842359; doi:10.1371/journal.pone.0082319)
Supplement: Table S3 — Spades De novo assembly statistics, multi-cell mode. (DOCX) [file pone.0082319.s009.docx]

**Table S3. Spades De novo assembly statistics, multi-cell mode^a^**

| Sample^b^ | Nb. contigs > 500bp | Aligned contigs | Unaligned bases (bp)^c^ | N50 of aligned contigs (bp) | Genome coverage (%)^d^ |
| --- | --- | --- | --- | --- | --- |
| control | 49 | 47 | 1302 | 200564 | 99.96 |
| cells2 | 63 | 59 | 6238 | 140990 | 99.97 |
| cells3 | 74 | 63 | 11905 | 116623 | 99.97 |
| cells4 | 116 | 96 | 30175 | 89238 | 95.32 |
| cells5 | 194 | 74 | 176800 | 41880 | 34.91 |
| gDNA1 | 48 | 48 | 0 | 173694 | 99.89 |
| gDNA5 | 70 | 51 | 19221 | 184768 | 99.97 |
| gDNA6 | 90 | 60 | 41566 | 173757 | 99.93 |
| gDNA7 | 214 | 105 | 183775 | 52884 | 96.50 |
| gDNA8 | 330 | 60 | 373486 | 30129 | 30.64 |

^a^ MDA samples were assembled *de novo,* and the resulting contigs were aligned to the *B.australis* reference sequence

^b^ The control sample correspond to the unamplified sample sequenced in the current study. All other samples correspond to MDA samples as detailed in Table 1 and 2.

^c^ The total length of contigs not which did not align to the *B.australis* reference sequence

^d^ The percentage of genome positions covered by at least one assembled contig
