# Supplementary material for: Testing the Reproducibility of Multiple Displacement Amplification on Genomes of Clonal Endosymbiont Populations
Source: PLoS One. 2013 Nov 27;8(11):e82319. doi: 10.1371/journal.pone.0082319 (PMC3842359; doi:10.1371/journal.pone.0082319)
Supplement: Table S4 — Genome recovery, contamination and chimeric reads, published data. (DOCX) [file pone.0082319.s010.docx]

**Table S4. Genome recovery, contamination and chimeric reads, published data**

| Sample | Sequencing method | Reads mapped (%) | Genome recovery (%)^a^ | Read length /insert size (bp) | % proper pairs/reads | % mapped on same strand | % mapped on opposite strands^b^ | Total non-proper reads/read-pairs (%) |
| --- | --- | --- | --- | --- | --- | --- | --- | --- |
| *B. australis* | Illumina, paired-end | 99.4 | 100 | 330 | 98.8 | 0.57 | 0.64 | 1.2 |
| *Wolbachia, w*No | Illumina, paired-end | 94.0 | 100 | 360 | 96.3 | 2.05 | 1.67 | 3.7 |
| *Wolbachia, w*Ha | Illumina, paired-end | 98.3 | 100 | 400 | 96.0 | 2.43 | 1.57 | 4.0 |
| *Wolbachia, w*No | 454 single-end | 98.7 | 98.7 | 340 | 98.8 | NA | NA | 1.2 |
| *Wolbachia, w*Ha | 454 single-end | 96.7 | 97.7 | 360 | 98.9 | NA | NA | 1.1 |
| *M. mitochondrii* | 454 single-end (GS-FLX) | 98.6 | 98.6 | 250 | 97.5 | NA | NA | 2.5 |
| *M. mitochondrii* | 454 single-end (titanium) | 99.1 | 99.8 | 340 | 97.0 | NA | NA | 3.0 |
| *Wolbachia, w*No | 454 paired-end | 96.7 | 98.2 | 2600 | 85.3 | 9.13 | 5.58 (1.24, 4.34) | 14.7 |
| *Wolbachia, w*Ha | 454 paired-end | 99.4 | 96.8 | 2700 | 86.4 | 8.56 | 5.08 (0.99, 4.09) | 13.6 |
| *M. mitochondrii* | 454 paired-end | 67.7 | 90.0 | 2300 | 67.1 | 23.1 | 9.75 (4.34, 5.41) | 32.9 |

^a^ The percentage of genome positions with at least one mapped read (see methods)

^b^ For the 454 paired-end data, the percentage of reads mapped facing outwards and reads mapped too far apart from each other are detailed in parenthesis
